# Malaria in children under-five: A comparison of risk factors in lakeshore and highland areas, Zomba district, Malawi

**DOI:** 10.1371/journal.pone.0207207

**Published:** 2018-11-12

**Authors:** Precious L. Hajison, Shingairai A. Feresu, Bonex W. Mwakikunga

**Affiliations:** 1 *Preluha consultancy, Namiwawa street, Newroad location, Zomba, Malawi; 2 University of Pretoria, Faculty of Health Sciences, School of Health Systems and Public Health, Epidemiology & Biostatistics Track, Pretoria, South Africa; 3 University of Fort Hare, Faculty of Health Sciences, East London, South Africa; 4 DST/CSIR Nanotechnology Innovation Centre, National Centre for Nano-Structured Materials, Council for Scientific and Industrial Research, Pretoria, South Africa; Universidade Nova de Lisboa Instituto de Higiene e Medicina Tropical, PORTUGAL

## Abstract

**Background:**

In Malawi, children under the age of five living in different geographical areas may experience different malaria risk factors. We compare the risk factors of malaria experienced by children under the age of five from Zomba district, who reside in lakeshore and highland areas.

**Methods:**

We conducted a case control study of 765 caregivers, cases being children under-five who were diagnosed with malaria, and obtained matched controls from local health facilities and communities. We used a multivariate logistic regression to identify individual and household risk factors.

**Results:**

In lakeshore areas, risk factors were households located one kilometer or less away from stagnant water (AOR: 2.246 95% CI: 1.269 to 3.975 P-value: 0.005); or if the household had obtained a mosquito bed net more than one year ago (AOR: 1.946 95% CI: 1.073 to 3.529 P-value: 0.028). In highland areas, risk factors were households which used a borehole/unprotected well (AOR: 1.962 95% CI: 1.001 to 3.844 P-value 0.050), communal standpipe (AOR: 3.293 95% CI: 1.301 to 8.332 P-value 0.012), and un-protected dug well in their yards (AOR: 16.195 95% CI: 2.585 to 101.464 P-value 0.003) as their drinking water sources. In highland areas, caregivers not attending health talks on malaria prevention messages was a risk factor (AOR: 2.518 95% CI: 1.439 to 4.406 P-value: 0.001).

**Conclusion:**

Children under the age of five living in highland areas experience different malaria risk factors compared to children living in lakeshore areas. Settling away from stagnant/open water source in lakeshore and encouraging caregivers to attend health talks on malaria prevention in highlands can help reduce malaria transmission. Nevertheless, using a mosquito bed net that is more than one year old is a common risk factor in both locations. Using new mosquito bed nets can significantly reduce the risk of contracting malaria in children under the age of five.

## Introduction

Malaria is a major cause of morbidity and mortality in Malawi and many other countries world-wide [1]. In Malawi, the burden of malaria in children under the age of five was estimated at 24% in 2017 [2]. Malaria is estimated at 34% among children whose mothers have no formal education [2], and causes 30% of outpatient visitation in children younger than 5 years old [3]. Recently, 95% of Malawi’s population was reportedly at risk of malaria [4]. The Zomba district of Malawi is classified as an endemic malaria area [5].

The government of Malawi distributes free Insecticide Treated Bed Nets (ITNs) to people living in rural and malaria prone areas to achieve and maintain universal coverage. Universal coverage of ITNs is defined as ownership of at least one net per two people living in a household [2, 6]. According to the Malawi Demographic Health Survey (MDHS) of 2010, the coverage of children under the age of five sleeping under mosquito bed nets has steadily increased from 55.4% in 2010 [7] through 67% in 2014 [8] and up to 68% by 2017 [2]. Despite malaria prevention efforts focusing on children younger than five and wide coverage with ITNs, new malaria cases are still occurring [9, 10]. To prevent malaria in this age group, we need to identify relevant risk factors.

Previous studies in Malawi, reported a number of malaria risk factors. A study done in Blantyre by Walldorf *et al* [11] reported that type of household and education level (secondary or above) of a household head reduced the risk of contracting malaria. A case control study done in Blantyre used a multivariate spatial logistic model to identify that children who visited rural areas were more at risk of contracting malaria than those who do not visit rural areas [12]. Additionally, the study revealed that younger children living in poor households had a higher risk of contracting malaria [12]. A study looking at malaria and anemia in antenatal women in Blantyre, Malawi revealed that malaria transmission was higher in the rainy season, in women younger than 20 years, and in the first and second trimester of pregnancy [13]. Similarily, Walldorf *et al* [11] compared seasonal risk factors of malaria and found that school going children had higher risk of contracting malaria in the rainy season compared to dry season. Other risk factors for malaria revealed in the study include lower education level, not sleeping under bed nets and not using Indoor Residual Spraying (IRS). Mathanga *et al* [14] compared malaria risk factors in urban and peri-urban areas in Malawi. Univariate analysis indicated that lower risk was associated with possession of a TV, electricity in the household and having a higher education. People living in urban and peri-urban areas had the same risk factors associated with contracting malaria [14]. Zgambo *et al* [15] compared malaria risk factors identified in the 2012 and 2014 Malaria Indicator Survey (MIS) and found that older children and those living in poor households were associated with contracting malaria in both 2012 and 2014 [15].

In other countries, a number of studies [16–20] compared malaria risk factors in relation to geographic location. Dev *et al* [16] conducted a study in Indian villages and found that malaria vectors were equally abundant in different types of settlements. In Tanzania, Ball *et al* [17] investigated the effect of topography in relation to risk factors of malaria, and found that high altitude reduced the risk of malaria transmission [17]. Attenborough *et al* [20] reported similar patterns in the north-western interior of Papua New Guinea. A study done in Ethiopia assessed the impact of constructing microdams on malaria incidence in relation to altitude [18]. Ghebreyesus *et al* [18] reported that the risk of malaria transmission was higher at lower altitudes. Although malaria transmission is perennial in lowland areas, Bødker *et al* [19] reported that the effects of altitude on vector numbers and parasite development is being negated by changing agricultural practices in the highlands of Tanzania.

At present, no studies have compared malaria risk factors by geographic location in Malawi, especially for children younger than five years old. Yet, the epidemiology of malaria has been linked to altitude of the area [19, 20]. Using a case control design, we assessed the risk factors of malaria in children under-five years living in the Zomba district in Malawi who lived in either lakeshore or highland areas. Identifying unique risk factors in different areas can help to target malaria prevention programs.

## Materials and methods

### Study setting

**Fig 1:**
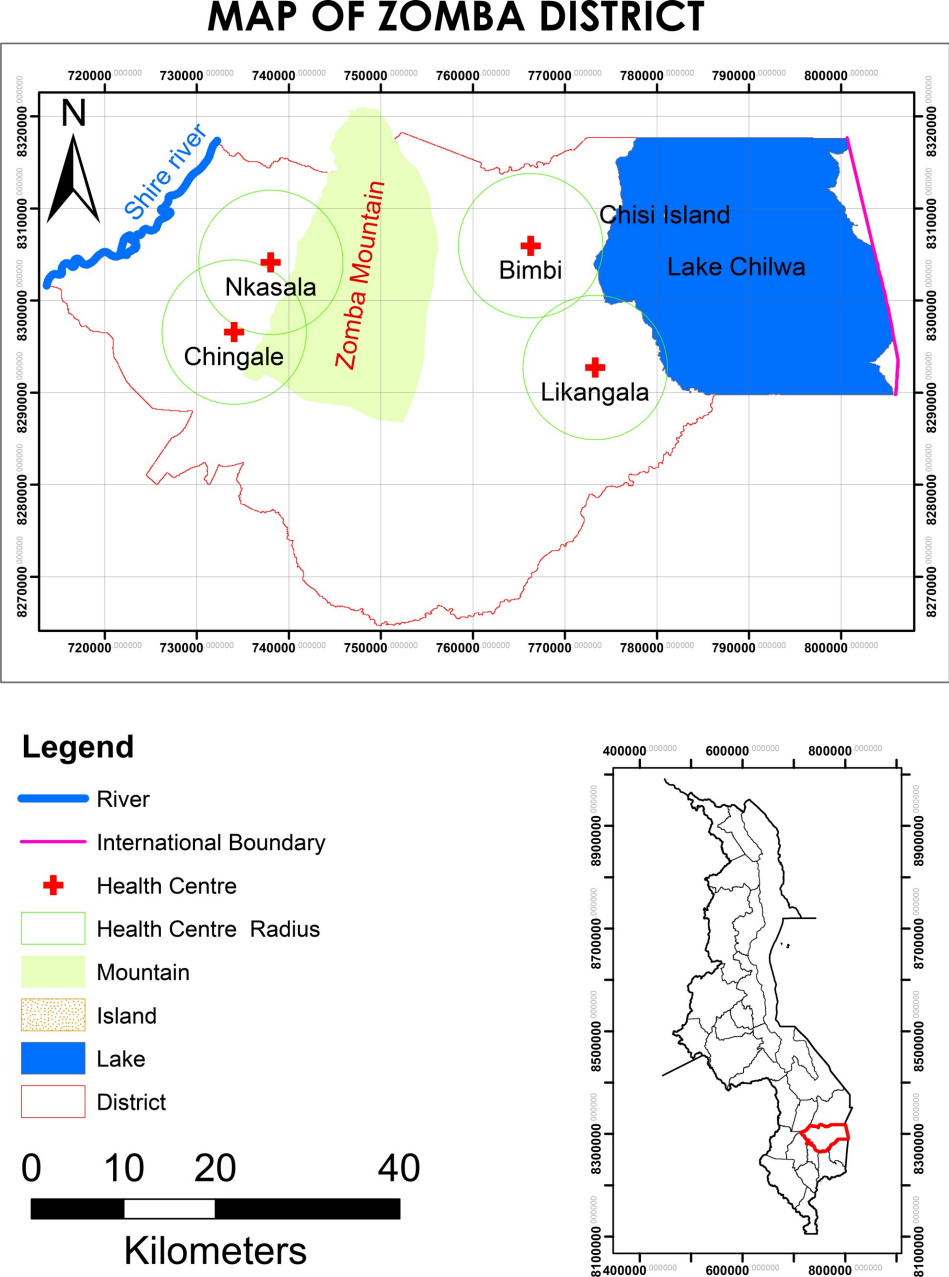
Zomba district map (study area). Maps of study area; Zomba district showing sites of the study: Highlands region, Lakeshore region with a catchment area of 8 km radius for each study site.

Our study area was the Zomba district (Latitude -15.3833, Longitude 35.3333 N -15 22′ 60′′, E 35 19′ 60′′) in southern Malawi (Fig 1) [21]. The Zomba district comprises of Lake Chirwa in the east and borders with the Shire River to the far west. The Zomba mountain range, and Zomba Mountain, is located between the Shire River and Lake Chirwa. We compared malaria risk factors of highlands and lakeshore areas. Lakeshore areas were areas within an eight-kilometer radius along the Shire River and Lake Chirwa. Highland areas were areas within an eight-kilometer radius along the Zomba mountain range (Fig 1). The areas between the Shire River and Zomba mountains, and between Lake Chirwa and Zomba mountains, were considered a buffer region and were excluded from the study.

Zomba health systems have 34 health facilities, including private, public and Christian Health Association of Malawi (CHAM). In this study we concentrated on public and CHAM health facilities. Public health facilities offer free care while CHAM facilities charge a contributory fee for health care services but they are relatively affordable and attract a good number of people of all kinds. Aside from being expensive, private health facilities are located in the town of Zomba, which is in the buffer region and thus excluded from the study. Seven health facilities were eligible in the lakeshore area, while six facilities were eligible in the highland area. Of the eligible facilities, CHAM and public facilities were grouped together for random selection in their respective stratums. According to each stratum (lakeshore and highland), simple random sampling was done and two facilities were picked from each stratum. Four health facilities included three public health facilities and one CHAM facility, of which Mkasala and Chingale health centers were located in the highlands, and Bimbi and Likangala health centers were located in the lakeshore area.

### Study design

In our study, cases were children under-five who tested malaria positive using malaria rapid diagnostic test (mRDT) during a health facility visit. Each case was matched to a control for under-five child. Controls comprised two groups; namely health facility and community based controls. Health facility controls were children who tested negative for malaria, but were diagnosed with other infections, at the same health facility. Community based controls were children who were randomly selected by visiting the village of a malaria case. Community based controls were children under-five who tested malaria negative with mRDT and had axillary temperature of less than 37.5°C.

### Sample size and sampling technique

To determine if malaria risk differed between lakeshore and highland areas for children younger than five years old in Zomba district, Malawi. According to Dzinjalamala [22] malaria in under-five-year children accounts for 60.2% in Malawi, however during the wet season, the prevalence increases depending on geographic location. Considering these estimates, we expected that 45% of under-five-year children would be infected by malaria in the highland area, and about 60% of under-five children would be infected by malaria in the lakeshore area. The normal distribution of two sided confidence level was set at 95% while the normal distribution corresponding to power^1^ was at 90%.

We used a ratio of 1 case to 2 controls, and standard error of 0.05 to estimate a suitable sample size. For every case enrolled in the study, we recruited two controls from the same catchment area. This ensured that each health facility recruited equal numbers of cases and controls. After computing this in Epi-Info, we had a total sample size of 765 children under-five years according to Fleiss w/CC; 255 cases, 255 hospital based controls, and 255 community based controls. At each health facility, the study team worked in the outpatient department and identified caregivers of under-five children. After children and caregivers received care, they were invited to participate in the study. If the caregiver agreed to participate, a research assistant conducted a screening to determine if the participant was eligible or not. Participants were excluded if malaria was diagnosed using symptoms alone without a lab confirmation test, those who were severely sick and were referred for tertiary care, and those who were already interviewed for the same study. From the villages where the malaria case lived, we also enrolled community based controls by first contacting the village head, who identified the households with under-five children in the village. From the list, the research assistant randomly sampled the households that had an under-five child. If the village had no under-five children, the next closest village was considered until a matching control was found. The caregiver of the selected under-five child was invited to participate in the study. Since other children act as a reservoir for malaria, upon acceptance of the caregiver, a trained Lab Technician/nurse conducted a confirmatory mRDT, using similar SD Bioline malaria Ag P.f/pan (HRP-II) ^TM^ which was also used in the health facilities in to diagnose malaria. If the child tested malaria negative and their temperature was less than 37.5°C, then the caregiver was recruited as a community control. Those children, who had suspected clinical malaria, but tested malaria negative, including children who had been tested or treated for malaria in the past two weeks were excluded as controls. Children who had suspected malaria and tested positive were referred to the nearest health facility for proper management.

### Data collection

We recruited four research assistants, two who worked in the health facility and two who worked in the community. The two community based research assistants were certified lab technician/nurse, who could conduct mRDT tests. All the research assistants completed a two day training course on the data collection tool.

Before data collection started, we piloted the data collection tool for validation. We selected one health facility (Matawale health facility) and conducted 15 interviews, five with caregivers of under-five children with malaria in the outpatient department; five with caregivers of under-five children not diagnosed with malaria (hospital based controls), and five with caregivers in the community (community based controls). After the pilot, we modified all the questions that were difficult to ask and those which respondents struggled to understand.

Data were collected using structured questionnaires to interview consenting caregivers of children under-five who were either cases, hospital or community based controls. Caregivers self-reported on ownership of a bed net (yes/no), attended health talks on malaria prevention (yes/no), how far they stayed from the nearest river stream or stagnant water (<1km/ >1km). Caregivers self-reported on the main material of the dwelling house (cement blocks, stone with lime, mud or sand, burnt bricks), type of the floor for the dwelling (floor of earth or sand, floor of palm/bamboo, cemented floor); sanitation of the household compound (clear weeds as soon as they grow/clear weeds once a month/clear weeds once a year/ never clear the weeds); literacy level of the household heads (No formal education/primary school/secondary school/ college or university), main water source for the compound (piped water/unprotected well/protected well/river or lake or stream); knowledge of antimalarial prescription, assessed if the respondents were able to pick the correct dose and frequency. We assessed knowledge of antimalarial prescription by asking the caregiver to give the standard prescription dose of antimalarial (LA) used to treat uncomplicated malaria in Malawi. If caregivers gave the correct dose, they were coded as having knowledge of antimalarial prescription and vice versa. We assessed the preventive measures used (use of bed net/burn the dung/taking antimalarial prophylaxis/use of mosquito repellent).

Data were collected over five weeks, starting from January 2016 to February 2016.

The study was conducted in strict accordance with the principles of the Declaration of Helsinki. The protocol was approved by University of Pretoria Ethics Committee, South Africa and National Commission for Science and Technology (NCST) review board, Malawi, under references 485/2015 and NCST/RTT/2/6 respectively. The Zomba district health office in Malawi permitted the collection of data. Additionally, we obtained written informed consent from the caregivers who were the legal guardians of eligible under-five children (cases and controls) before conducting exit interviews.

### Statistical analyses

In the analysis, our response variable was a binary (y = 1 if case or 0 if control). We used Chi-square tests of association to examine the following explanatory variables to determine risk factors: economic status of the household including type of the dwelling houses; sanitation of the household compound; literacy level of the household heads, and main water source for the compound; the knowledge of how to administer prescribed antimalarial drugs for non-complicated malaria and preventive measures; malaria prevention health education talks; possession, and usage of bed nets; gender of the child and the caregiver.

To compare the risk factors between children living in lakeshore and highland areas, we stratified data by geographic area and analyzed the risk factors separately for each area. We used summary statistics and frequency tables to quantify and describe the data. We used univariate and multivariate logistic regression analyses to model the effect of risk factors on malaria in the under-five children. For the multivariate regression models, we pre-selected potential explanatory variables if the univariate model indicated significance using a relaxed p-value of ≤ 0.25. We checked all the explanatory variables for co-linearity, and when a pair of co-linear variables were identified, then one of the two was dropped from the model. We tested all potential interactions for significance, and excluded all non-significant interactions from the model. We included all candidate explanatory variables in a saturated model, and removed non-significant variables in a stepwise fashion until we reached the final model.

## Results

Table 1 presents demographic and descriptive characteristics of the study population. Generally more female caregivers took sick children to hospital, compared to male caregivers across both geographical locations. More female children in lakeshore visited health facilities, compared to male children, while in highland there was not much difference in gender of the children who sought health care. Table 1 shows that about two thirds of the household heads in lakeshore had primary school education only, while in highlands, less than half of the household heads had primary school education only. In the highlands, many more household heads had secondary education compared to the lakeshore.

**Table 1:** Demographic characteristics of respondents in case control study in lakeshore and highland areas in Zomba District, Malawi, 2016.

Variable Name	Highland					Lakeshore				
	Cases		controls			Cases		Controls		
	(n = 126)	%	(n = 254)	%	P-Value	(n = 129)	%	(n = 256)	%	P-Value
**Respondent gender**					**0.315**					**0.004**
Male	15	11.90	22	8.66		7	5.43	2	0.78	
Female	111	88.10	232	91.34		122	94.57	254	99.22	
**Gender of <5 child**					**0.512**					**0.819**
Male	59	46.83	128	50.39		56	43.41	108	42.19	
Female	67	53.17	126	49.61		73	56.59	148	57.81	
**Health facility**					**0.827**					**0.829**
Mkasala H C	64	50.79	126	49.61						
Chingale H C	62	49.21	128	50.39						
Likangala H C						64	49.61	130	50.78	
Bimbi H C						65	50.39	126	49.22	
**Education level of HH**					**0.213**					**0.411**
No education	16	12.70	28	11.02		15	11.63	18	7.03	
Primary school	59	46.83	110	43.31		86	66.67	188	73.44	
Secondary school	51	40.48	108	42.52		27	20.93	48	18.75	
College	0	0.00	8	3.15		1	0.78	2	0.78	

Malaria risk factors in the lakeshore areas were quantified using logistic regression to calculate univariate and multivariate odds ratios (Table 2). According to univariate analysis, significant odds ratio for malaria in lakeshore areas in children under-five were; having cemented floor inside the house (OR: 3.529 95% CI: 1.014–12.284, p = 0.048) over those households with earth or sand, households which did not have the mosquito bed net to use during sleeping (OR:2.571 95% CI: 1.316–5.021, p 0.006) over those households with at least a mosquito bed net to use; household 1km or less away from the stagnant water (OR:1.759 95% CI: 1.095–2.824, p = 0.019) over households more than one kilometer away from a stagnant water source, if the household was using a mosquito bed net obtained more than one year ago (OR:1.771 95% CI: 1.035–3.030, p = 0.037) over households with mosquito bed nets obtained less than a year ago, and the risk increases as the period of time increases.

**Table 2:** Crude and adjusted odds ratios for malaria risk factors for children under-five in the lakeshore area of Zomba, Malawi, 2016. The dummy predictor variable being malaria status, and normative reference group being those people who were not diagnosed with malaria.

	Univariate Analysis: Crude odds ratio 95% Confidence Intervals			Multivariate Analysis: Adjusted odds ratio 95% Confidence Intervals		
Variable name	OR	95% CI	P-value	AOR	95% CI	P-value
Main material of the floor inside the house						
Earth/Sand	1.0			1.0		
Cement	3.529	1.014–12.286	0.048	3.117	0.793–12.249	0.104
Knowledge of antimalarial prescription by the caregiver						
No	1.0			1.0		
Yes	6.565	3.768–11.436	0.001	6.959	3.683–13.149	0.001
Distance of the household from the stagnant water						
More than a kilometer away from the yard	1.0			1.0		
1 kilometer or less away from the yard	1.759	1.095–2.824	0.019	2.246	1.269–3.975	0.005
Period in Month household obtained mosquitoes bed net						
One year or less	1.0			1.0		
Above one year up to 35 months	1.771	1.035–3.030	0.037	1.946	1.073–3.529	0.028
36 months above	2.559	1.381–4.741	0.003	2.295	1.139–4.624	0.020
Since obtaining the bed net was it soaked to protect from malaria						
Yes	1.0			1.0		
No	1.333	0.661–2.689	0.421	1.763	0.789–3.943	0.167
Sex of the child						
Female	1.0					
Male	7.287	1.492–35.599	0.014			
Main material of the external/outer wall						
Bricks	1.0					
Cane/palm/trunk	1.933	0.259–14.413	0.520			
Mud/Sand	0.957	0.578–1.583	0.864			
What is the main material of the roof?						
Galvanized sheet	1.0					
Thatch/Palm leaf	1.136	0.631–2.047	0.671			
What type of toilet facility do you have in the household						
Pit latrine with slab	1.0					
Traditional pit latrine	2.571	0.957–6.911	0.061			
With no latrine	2.399	0.515–11.185	0.265			
Highest level of education of the household head						
No Education	1.0					
Primary school	0.549	0.264–1.140	0.108			
Secondary school	0.675	0.294–1.551	0.108			
College	0.600	0.049–7.283	0.688			
Can the household head fluently read?						
With difficulties in Chichewa or Cannot read at all	1.0					
Fluent read Chichewa only	0.669	0.367–1.219	0.189			
Fluent read English and Chichewa	0.825	0.464–1.465	0.511			
Chichewa yes but difficulties in English	0.851	0.417–1.738	0.658			
Source of drinking water						
Public unprotected well, lake/river/stream	1.0					
Borehole	1.637	0.639–4.191	0.304			
Communal standpipe	4.583	1.263–16.635	0.021			
Ever attended health talks on malaria prevention						
No	1.0					
Yes	1.699	0.855–3.378	0.130			
Professional cadre conducted the health talks						
Nurses	1.0					
Health Surveillance Assistants HAS	1.019	0.529–1.959	0.955			
Community leaders/radio/ Television	0.469	0.048–4.554	0.514			
How often did you hear malaria prevention messages						
More than once a month	1.0					
Once a month	1.015	0.649–1.586	0.947			
Once in three months or above	1.218	0.561–2.644	0.617			
How often did you attend malaria prevention messages?						
More than once a month	1.0					
Once a month	1.107	0.676–1.812	0.687			
Once in three months and above	0.756	0.289–1.977	0.569			
Never attended	0.563	0.257–1.231	0.150			
For how long can it take for you to access the preventive messages of malaria						
Less than 1 kilometer	1.0					
1 kilometer and above	0.991	0.513–1.917	0.979			
How often do you clear the weeds at your yard						
Once a month or never	1.0					
As soon as they grow	0.236	0.053–1.044	0.057			
What is the most important way of preventing Malaria?						
Sleeping under the net	1.0					
Burn the dung	2.012	0.278–14.363	0.491			
Household having mosquito bed net to be used						
Yes	1.0					
No	2.571	1.316–5.021	0.006			
How many mosquito nets does your house hold have?						
Three or more	1.0					
Two bed nets	0.729	0.249–2.137	0.565			
One bed net	0.987	0.356–2.732	0.980			
What is the shape of the mosquito net						
Conical	1.0					
Rectangle	0.906	0.163–5.023	0.910			
Means of obtaining the mosquito bed net						
Bought the bed net	1.0					
Received for free from government hospital	1.335	0.419–4.244	0.625			
Received for free from another NGOs	2.625	0.574–11.998	0.213			
Obtaining the bed net together with treatment kit or LLIN						
Yes	1.0					
No	1.234	0.553–2.752	0.608			
Period in months was the bed net get treated						
Within twelve months or less	1.0					
Thirteen up to 23 months	0.833	0.061–11.277	0.0891			
Twenty four month or above	2.813	0.502–15.768	0.240			
The reason why mosquito bed net not treated						
Did not have money to buy the treatment kit	1.0					
Nowhere to buy treatment kit	0.264	0.056–1.251	0.093			
It is long lasting treated net	0.842	0.499–1.523	0.630			
Any person including the under-five slept in the mosquito bed net previous night						
Yes	1.0					
No	3.371	0.555–20.476	0.187			
What shape of mosquito net do you prefer?						
No preference	1.0					
Conical	0.884	0.382–2.049	0.774			
Rectangle	0.648	0.313–1.342	0.243			

According to the multivariate analysis, the following risk factors were statistically significant in the lakeshore area (Table 2). Children under-five residing in households located one kilometer or less away from the stagnant water had 2.246 increased odds of having malaria than those who reside more than a kilometer away from stagnant water. Children under-five with caregivers who knew the correct number of days for administering uncomplicated antimalarial drugs had 6.959 increased odds of contracting malaria. In the lakeshore area, children under-five who were using mosquito bed nets which were obtained more than one year ago had 1.946 increased odds of contracting malaria than those who were using mosquito bed nets obtained less than a year ago.

Malaria risk factors in the highland areas were identified in the similar manner as done for the lakeshore area, according to the univariate analysis (Table 3) the following odds ratios were reported. Those households which received their mosquito bed net for free from NGOs (OR: 8.615 95% CI: 1.576–47.091, p = 0.013) over households who had bought their mosquito bed nets. Those households who obtained their bed net more than one year ago (OR: 2.129 95% CI: 1.105–4.101, p = 0.024) over households who had obtained bed nets less than a year ago; and if the household did not re-treat their bed nets because of using a long lasting insecticide treated mosquito ben nets (LLIN) (OR: 2.161 95% CI: 1.035–4.514, p = 0.040) over those who did not have money to buy treatment kits.

**Table 3:** Crude and adjusted odds ratios for malaria risk factors for children under-five in highland areas of Zomba, Malawi, 2016. The dummy predictor variable being malaria status, and normative reference group being those people who were not diagnosed with malaria.

	Univariate Analysis: Crude odds ratio 95% Confidence Intervals			Multivariate Analysis: Adjusted odds ratio 95% Confidence Intervals		
Variable name	OR	95% CI	P-value	AOR	95% CI	P-value
Main material of the external/outer wall						
Bricks	1.0			1.0		
Mud/Sand	0.358	0.191–0.669	0.001	0.335	0.168–0.670	0.002
Bamboo with Mud/cardboard	1.486	0.757–2.920	0.250	1.185	0.569–2.466	0.650
Stone with Lime/cement blocks	0.735	0.430–1.254	0.259	0.860	0.479–1.545	0.615
Source of drinking water						
Public unprotected well, lake/ river/ stream	1.0			1.0		
Borehole/protected well	1.528	0.824–2.834	0.179	1.962	1.001–3.844	0.050
Communal standpipe	2.415	1.035–5633	0.041	3.293	1.301–8.332	0.012
Unprotected dug well in the yard	7.812	1.379–44.234	0.020	16.195	2.585–101.464	0.003
Ever attended health talks on malaria prevention						
Yes	1.0			1.0		
No	2.742	1.650–4.555	<0.001	2.518	1.439–4.406	0.001
Period in Month household obtained mosquitoes bed net						
One year or less	1.0			1.0		
Above one year up to 35 months	2.129	1.105–4.101	0.024	1.977	0.956–4.087	0.066
36 months above	2.129	1.315–3.447	0.002	2.487	1.475–4.192	0.001
Sex of the child						
Female	1.0					
Male	0.867	0.565–1.329	0.513			
Main material of the floor inside the house						
Earth/Sand	1.0					
Cement	0.711	0.369–1.366	0.306ta			
What is the main material of the roof?						
Galvanized sheet	1.0					
Thatch/Palm leaf	1.266	0.774–2.071	0.348			
What type of toilet facility do you have in the household						
Pit latrine with slab/improved latrine	1.0					
Traditional pit latrine	0.909	0.421–1.963	0.808			
With no latrine	0.455	0.082–2.526	0.368			
Highest level of education of the household head						
No Education	1.0					
Primary school	0.939	0.470–1.873	0.857			
Secondary school/college	0.769	0.383–1.545	0.461			
Can the household head fluently read?						
With difficulties in Chichewa or Cannot read at all	1.0					
Fluent read Chichewa only	0.750	0.359–1.566	0.444			
Fluent read English and Chichewa	0.690	0.366–1.300	0.251			
Chichewa yes but difficulties in English	0.750	0.378–1.174	0.539			
Knowledge of antimalarial prescription by the caregiver						
No	1.0					
Yes	0.989	0.599–1.635	0.968			
Distance of the household from the stagnant water						
More than a kilometer away from the yard	1.0					
1 kilometer or less away from the yard	1.250	0.384–4.067	0.711			
Professional cadre conducted the health talks						
Nurses	1.0					
Health Surveillance Assistants HAS	1.187	0.509–2.764	0.691			
Community leaders/radio/ Television	1.200	0.2933–4.909	0.800			
How often did you hear malaria prevention messages						
More than once a month	1.0					
Once a month	0.663	0.408–1.076	0.096			
Once in three months or above	2.265	1.161–4.419	0.017			
How often did you attend malaria prevention messages?						
More than once a month	1.0					
Once a month	0.526	0.282–0.980	0.043			
Once in three months and above	0.564	0.327–0.971	0.039			
Never attended	2.368	0.641–8.739	0.196			
For how long can it take for you to access the preventive messages of malaria						
Less than 1 kilometer	1.0					
1 kilometer and above	0.578	0.368–0.908	0.017			
How often do you clear the weeds at your yard						
Once a month or never	1.0					
As soon as they grow	0.606	0.382–0.962	0.034			
What is the most important way of preventing Malaria?						
Sleeping under the net	1.0					
Burn the dung/mosquito coil	3.631	1.288–10.237	0.015			
Sleeping outside the house when it is hot	1.089	0.098–12.138	0.945			
Spraying the house/taking antimalarial prophylaxis	3.268	0.538–19.831	0.198			
Household having mosquito bed net to be used						
Yes	1.0					
No	2.583	0.681–9.790	0.163			
How many mosquito nets does your house hold have?						
Three or more	1.0					
Two bed nets	0.813	0.325–2.034	0.658			
One bed net	0.867	0.367–2.048	0.745			
What is the shape of the mosquito net						
Conical	1.0					
Rectangle	0.789	0.373–1.674	0.538			
Means of obtaining the mosquito bed net						
Bought the bed net	1.0					
Received for free from government hospital	1.125	0.565–2.239	0.737			
Received for free from another NGOs	8.615	1.576–47.091	0.013			
Other means not specified	2.051	0.531–7.917	0.297			
Obtaining the bed net together with treatment kit or LLIN						
Yes	1.0					
No	1.095	0.657–1.826	0.727			
Since obtaining the bed net was it soaked to protect from malaria						
Yes	1.0					
No	0.995	0.614–1.613	0.984			
Period in months was the bed net get treated						
Within twelve months or less	1.0					
Thirteen up to 23 months	0.259	0.029–2.274	0.223			
Twenty four month or above	0.566	0.220–1.452	0.236			
The reason why mosquito bed net not treated						
Did not have money to buy the treatment kit	1.0					
Nowhere to buy treatment kit	1.176	0.454–3.049	0.738			
It is long lasting treated net	2.161	1.035–4.514	0.040			
Did not want a treated net	.	.	.			
Any person including the under-five slept in the mosquito bed net previous night						
Yes	1.0					
No	1.036	0.469–2.288	0.930			
What shape of mosquito net do you prefer?						
No preference	1.0					
Conical	0.521	0.228–1.187	0.121			
Rectangle	1.091	0.470–2.529	0.839			

According to the multivariate logistic regression analysis the following risk factors were statistically significant in the highland areas (Table 3). Children under-five living in houses made of mud/sand in its external/outer wall had 0.335 odds of contracting malaria over those under-five children living in the houses made of burnt bricks in the external wall. The households which accessed their drinking water from a borehole (AOD: 1.962), communal standpipe (AOD: 3.293) and unprotected dug well within the yard (AOR: 16.195) over households who accessed drinking water from unprotected public wells, lakes, rivers or streams. Children under-five whose caregivers had never attended health talks on malaria prevention had 2.518 odds of contracting malaria over those whose caregivers ever attended health talks on malaria prevention. Similarly to those living in lakeshore, children under-five whose household obtained bed nets more than 36 months ago or more, had 2.487 odds of contracting malaria compared to those households that had obtained mosquito bed nets less than one year ago.

## Discussion

In our previous study [21] we reported that the lakeshore areas of Zomba district had higher rates of malaria transmission than the highland areas, but little is known about the different individual and household risk factors in these areas. Bridging this knowledge gap, may target interventions and help to create a malaria free environment in both lakeshore and highland areas of Zomba. Our case-control study revealed different risk factors for children under-five living in lakeshore and highland areas. Many of the risk factors are linked to socio-economic factors such as level of literacy and access to health care. Our analysis also revealed risk factors linked to behaviors such when households had obtained and used mosquito bed nets.

In lakeshore areas, knowledge of administering prescribed antimalarial drugs for non-complicated malaria suggest that children of caregivers who has the knowledge have a greater malaria risk. These findings are contradictory to Mathanga *et al* [14] who investigated malaria risk factors in Blantyre and reported that malaria risk can be attributed to illiteracy [14]. In principle, illiterate caregivers may not always be aware of malaria prevention methods [17, 23]. However, our finding may be suggestive of negligence of the caregiver who knows how to administer antimalarial drugs. Or caregivers may simply be more knowledgeable about medication due to the high prevalence of malaria in the area. The use of malaria prevention methods may also be linked to people’s perceptions of malaria, a factor not addressed in this study.

Our study showed that children under-five, who lived in lakeshore areas and lived close (<1km) to open water sources, such as rivers, swamps or unprotected wells had a higher malaria risk than those who lived far (>1km) from an open water source. Lakeshore areas are prone to flooding, which leads to more stagnant water bodies after the flooding period which eventually become ideal mosquito breeding habitats. Lakeshore areas are also usually flat, leading to the accumulation of stagnant water bodies. Many people living in lakeshore areas may fish for a livelihood, causing them to stay closer to water bodies, hence exposing themselves to malaria vectors.

We also found that the means of obtaining a mosquito bed net influenced the risk of contracting malaria in highland areas. Most Malawians use free distributed mosquito bed nets, and in our study, we found that children under five living in a household that owned a free bed net distributed by Non-Governmental Organizations (NGO) had an increased malaria risk compared to those who had bought their own mosquito bed net. Freely distributed mosquito bed nets [2] may be of poor quality and unable to prevent mosquito bites, due to ineffective insecticides. Often poor households are not able to replace old mosquito bed nets which also lose their efficacy. Both in highland and lakeshore areas, malaria risk was greater if caregivers had obtained mosquito bed nets prior to the past 12 months. This suggests that not retreating old bed nets, because they are LLINs, may have increased the risk of malaria in children under-five. As discussed above, LLINs may become less effective over time or become damaged.

This study also revealed that drinking water sources are a risk factor for contracting malaria in highland areas. Those caregivers who use unprotected dug wells in the yard, and those who use communal standpipes have a higher risk of contracting malaria. These water sources may harbor malaria vectors. The communal standpipe may lack control of water which spills over when used, water puddles may become stagnant and provide a conducive environment for mosquito breeding. Caregivers who use unprotected dug wells as a drinking water source probably live closer to these dug wells which increases their risk of being bitten by mosquitoes and contracting malaria.

In highland areas, access to malaria prevention messages at health facilities was an important malaria risk factor for children under-five [24]. The highland areas of Zomba are hilly and services are scattered limiting access to malaria prevention messages and health services. Additionally, travelling in hilly areas is always difficult, which may prohibit people from travelling long distances for the sole reason of attending health education talks on malaria prevention. Lakeshore areas are usually flat and easy to navigate from one point to another, talks on malaria prevention are thus widely available, and not an important risk factor for contracting malaria.

The pattern of malaria risk factors that emerged in this study, clustered according to geographic location, may be driven by other factors beyond those we examined. There may be delayed uptake of malaria prevention due to limited access to health care or distance to health facility, or contributory fee which is required in the CHAM health facilities.

### Study limitations

Malaria cases were diagnosed by mRDT. However, it is reported that, mRDTs sometimes fail to detect low density parasitaemia (<200 parasites/μl) [25]. Our study may also be limited by the self-reporting of the caregivers and subsequent recall bias, where caregivers may easily forget things that happened long ago. We tried to make sure that most of the questions did not require recollection from the distant past.

## Conclusion

We observed different variables associated with malaria diagnosis between highland and lakeshore regions, although there is a common risk factor shared across areas. These findings suggest that some interventions such as the use of new mosquito bed nets may help to reduce malaria risk among under-five children, and may be suitable to both geographic locations. People in lakeshore areas may potentially benefit by moving their households more than one kilometer away from water sources, whilst people living in highland areas should be encouraged to manage stagnant and open water sources close to their households and be encouraged to attend health talks on malaria prevention.

## Supporting information

S1 Questionnaire The Zomba malaria case control questionnaire-English & Chichewa. (DOCX)

S1 Dataset The Zomba malaria case control dataset. (DTA)
