# Supplementary material for: Malaria in children under-five: A comparison of risk factors in lakeshore and highland areas, Zomba district, Malawi
Source: PLoS One. 2018 Nov 12;13(11):e0207207. doi: 10.1371/journal.pone.0207207 (PMC6231663; doi:10.1371/journal.pone.0207207)
Supplement: S1 Questionnaire — (DOCX) [file pone.0207207.s001.docx]

**Structured questionnaire**

**Seasonality of Malaria epidemic in under-five-year old children in Lake Shore compared to Highland areas in Zomba district, Malawi**

| **IDENTIFICATION** | | | | | |
| --- | --- | --- | --- | --- | --- |
| **ID1** | **Respondent ID** |  | **ID2** | **TA Name** |  |
| **ID3** | **Respondent’s Name** | FIRST NAME | **ID4** | **Village Name** |  |
|  |  | SURNAME |  |  |  |
| **ID5** | **Respondent’s Sex** | 1 = Male  2 = Female | **ID6** | **Child sex** | 1 = Male  2 = Female |
| **ID7** | **District Name** |  | **ID8** | **Date of Interviews** | [____/_____/_________] |
| **ID 9** | **Malaria status** | 0 = control  1 = cases | **ID 10** | **Location** | 1 = Highland  2 = Lakeshore |

| 1 | What is the main material of the floor inside of your house?  *Kodi pansi panyumba yanu panapangidwa ndi chani kwenikweni* | 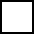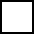 | 11=Earth/Sand/Dothi/mchenga  12=Dung/Ndowe  13=Wood Planks/Zipapati  14=Palm/Bamboo/Nsungwi/ bwazi  15=Parquet/Polised wood/polishi | 16=Vinyl/Asphalt strips  17=Ceramic Tiles/matailosi  18=Cement/simenti  19=Carpet/kapeti  98=Other/zina |
| --- | --- | --- | --- | --- |
| 2 | What is the main material of the exterior/ outer wall?  *Kodi khoma la nyumba yanu linapangidwa ndi chani kwenikweni?* |  | 00=No walls/ ilibe khoma  11=Cane/Palm/Trunks/bwazi/zipika  12=Mud/Sand/dothi/mchenga  13=Bamboo with Mud/yomata  14=Stone with Mud/yamiyala  15=Plywood  16=Cardboard/makatoni | 17=Reused wood /Nkhuni zogwiritsidwa kale ntchito  18=Cement/siment  19=Stone with Lime/Cement/ miyala ndi simenti  20=Bricks/Njerwa  21=Cement Blocks/ njerwa za simenti  22=Wood Planks/Shingles/ zipapati  98=Other/zina |
| 3 | What is the main material of the roof?  *Kodi denga la nyumba yanu linapangidwa kuchokera ku chani kwenikweni* |  | 00=No Roof/palibe denga  11=Thatch/Palm leaf/udzu/bwazi  12=Rustic Mat  13=Palm Bamboo/nsungwi  14=Wood Planks/zipapati  15=Cardboard/Makatoni  16=Galvanized sheet/Malata | 17=Wood/Nkhuni  18=Calamine/Cement Fiber  19=Ceramic tiles/Matiyilosi  21=Cement/simenti  22=Roofing shingles  98=Other/Zina |
| 4 | What type of toilet facility do you have in the household?  *Pakhomo pano muli ndi chimbudzi chamtundu wanji?* |  | 0=None / Palibe  1= Flush toilet/Chimbudzi cha madzi  2= Ventilated improved pit latrine/Chimbudzi chokumba chokhala ndi paipi | 3=Pit latrine with slab/Chimbudzi chokumba chokhala ndi silabu  4= Traditional pit latrine/chimbudzi chokumba  8=Other/zina  9=Don't Know |

| Q5 | What is the highest level of education of the household head attended?  *Kodi wamkulu wapakhomo pano analekezera pati Sukulu?* | Primary School ……………………………………………………………..1  Secondary School ………………………………………………………..2  College…………………………………………………………………………...3  No Education at all /sanaphunzire …………………………………………………….….4 |
| --- | --- | --- |
| Q6 | Can the household head fluently read?  ***Kodi wamkulu wapakhomo pano akhonza kuwerenga bwinobwino/mosajejema?*** | Cannot read at all/Sawerenga nkomwe…………………………………………………….…….0  Fluent readChichewaonly/Amawerenga bwino Chichewa chokha…………….…….1  Fluent read English and ChichewaAmawerenga bwino Chizungu ndi Chichewa…………………………………………………………………………………………………………2  Chichewa yes but with difficulties in English/Chichewa inde koma amavutika ndi Chizungu…………………………………………………………………………………………………...3 |
| Q7 | When you are prescribed LA by the physician, how long are you expected to take the drugs?  *A dokotala akakulemberani LA, mumayembekezera kumumwa nthawi yayitali bwanji?* | One day/Tsiku limdzi……………………………………………….………………..………………1  Two days/Masiku awiri…………………………………………………………………………..…2  Three days/Masiku atatu………………………………………………………………………..…3  Four days/Masiku anayi…………..……………………………………………………………..…4  Seven days/Masiku asanu ndi anayi…………………………………………….………...….5  Don’t know/Sakudziwa………………………………………………………………………..…….9 |
| Q8 | How far are you from the pond, stream/river or stagnant water?  *Kodi inuyo mwatalikana bwanji ndi dziwe, khwawa/mtsinje kapena madzi wodikha?* | It is within our yard/Lili pakhomo pompano……………………………….…………………..……….1  100 meters away from our yard/Lili pa mtunda wa ma mita 100……………………....…….2  1 kilometer away from our yard/Lili pa mtunda wa 1 kilomita………………..………..………3  More than a kilometer away from our yard/Lili pamtunda wopitilira 1 kilomita……...4 |
| Q9 | What is the main source of drinking water for members of your household?  *Kodi anthu apakhomo pano nthawi zambiri madzi akumwa amatunga kuti?* | Piped water - into dwelling /Mpopi wanyumba ...........................................1  Piped water – into yard/plot/Mpopi wapakhomo.........................................2  Communal standpipe/Mpopi wammudzi......................................................3  Borehole/Mjigo..............................................................................................4  Tanker truck/Tankala ...................................................................................5  Public well – unprotected/Chitsime chosatetezeka................................6  Public well protected/Chitsime chotetezedwa cha antu onse..............7  Dug well in yard/compound unprotected/chitsime chosatetezeka chapakhomo...................................................................................................8  Dug well in yard/compound –protected/Chitsime chosatetezedwa chapakhomo....................................................................................................9  river/stream/Mtsinje/Khwawa........................................................................10  Other/Zina…………………………………….…………………………..………………………..…….11  Don't know/Sakudziwa..................................................................................99 |
| Q10 | Have you ever attended the health talks on malaria prevention either in your village or at health centre?  Kodi munakhalapo pazokambirana zaumoyo zokhudza kupewa Malungo mmudzi mwanu kapena kuchipatala? | Yes/inde…………………………………………………………………………….…………….…1  No/Ayi……………………………………………………………………………………………...2  Don’t remember/Sakukumbukira…….………….........................................................3 |
| Q11 | Who conducted the health talks  Ankapangitsa zokambiranazo ndi ndani? | Nurse or clinician/ A nesi kapena adokotala………………………………………………….…..…..1  HSA in our village/Mlangizi wazaumoyo………………………………………….…….……………...2  Community leader at our village/ Mtsogoleri wammudzi kumudzi kwathu……………3  on radio or TV or read on posters/ Pa wailesi kapena kuwerenga pamapositala…….4 |
| Q12 | How often did you hear or attend malaria prevention messages?  Ndi mowirikiza bwanji pomwe munamva mauthenga okhudza kupewa Malungo kapena kukhala nawo pamauthenga okhudza kupewa Malungo | Every day lonse/Tsiku liri…………………………………………………………………………..….….1  Once a week/ Kamodzi pa sabata…………………………………………………………………..…2  More than once a month/Kuposera kamodzi pamwezi………………………………….…3  Once a month/Kamodzi pamwezi………………………………………………………….….….……4  Once in three months/Kamodzi pa miyezi itatu………………………………………….….…..5  At least once in a year/Kamodzi pachaka……………….…………………………………….…...6 |
| Q13 | For how long can it take for you to access the preventive messages of malaria  Kodi zikhonza kukutengerani mtunda wautali bwanji kuti mukapeze uthenga wokhudza kupewa kwa Malungo? |  |
| Q14 | How often do you clear the weeds at your yard?  Kodi pakhomo panu mumalambula mowirikiza bwanji? | Once a month/ Kamodzi pamwezi……………………………………………………………………………1  As soon as they grow/Akangomera………………………………………………………..………..………2  The yard is big therefore we clear one side and leave the other side/Bwalo ndilalikulu choncho timalambula mbali imodzi ndikusiya mbali ina…………………………………………..3 |
| Q15 | What is the most important way of preventing Malaria?  Kodi njira yofunikira kwambiri yopewera Malungo ndi iti?  **INTERVIEWER: READ THE OPTIONS** | Sleeping under the net/kugona mumasikito audzudzu……………………………………….1  Burn the dung/kuotcha ndowe………………………………………………………………….……….2  Sleeping outside house when it is hot/kugona panja kukamatentha………………….3  Taking LA beforehand/kumweratu LA usanadwale…………………..…………………….…..4  Spraying the house with mosquito repellent/Kupopela nyumba ndi mankhwala a udzudzu……………………………………………………………………………………………….…….…………5 |

| NO | QUESTIONS AND FILTERS | CORDING CATEGORIES | SKIP |
| --- | --- | --- | --- |
| Q16 | **Does your house hold have any mosquito nets that can be used while sleeping?**  **Kodi khomo lanu liri ndi masikito audzudzu omwe akhonza kugwiritsidwa ntchito pogona?** | YES-/Inde……………………………. 1  NO-/Ayi………………………....... 2 | >>26 |
| Q17 | **How many mosquito nets does your house hold have? –**  **Khomo lanu liri ndi masikito angati?** | NUMBER OF NETS- |  |

| Q18 | What is the shape of the mosquito net-  Masikitiwo ndiwotani/ ndi owoneka bwanji? | Conical /Yozungulira………..……………………..1  Rectangle-/ Yamakona……………………………2 | |
| --- | --- | --- | --- |
| Q19 | **How many months ago did your household obtain the mosquito net?**  **Papita miyezi ingati kuchokera pomwe khomo lanu linapeza masikitowo?**  *IF LESS THAN 1 MONTH RECORD 00* | Months ago/Miyezi  More than 36 months ago/ kuposera zaka zitatu………*95*  Not sure/Sakutsimikiza……………………………..….98 | |
| Q20 | **Did you buy the net or got it free of charge?**  **Kodi masikitowo munachita kugula kapena munalandira aulere?** | yes bought the net/ inde ndinachita kugula..………….1  no received for free from hospital/ Ayi ndinapasidwa mwaulele kuchokela kuchipatala.…………………...….2  No received for free from another NGO/ Ayi ndinapasidwa mwaulele kuchokela ku mabungwe……..3  Can’t remember/ Sindikukumbukila….……………..…4 | |
| Q21 | **When you got or receive the mosquito net, did it come with a treatment kit?**  **Mutagula kapena kulandira Masikitowa kodi anabwera ndi mankhwala ake?** | Yes/Inde …………………………………….……….…1  No/Ayi …………………………………..………..……2  Not sure/Sakutsimikiza ………………………….….…3 | |
| Q22 | **Since you got the mosquito net, was it ever soaked or dipped in a liquid to kill mosquitoes?-**  **Kuchokera pomwe munapeza masikitowo kodi ananyikidwako mmankhwala opha udzudzu?** | Yes/Inde……..………………………..………1  No/Ayi ……..………………………………….2 >>Q 20  NOT SURE /Sakutsimikiza……………………3 | |
| Q23 | **How many months ago were it soaked or dipped (re-treated)?**  **Papita miyezi ingati kuchokera pomwe inanyikidwa mmadzi?**  IF LESS THAN ONE MONTH RECORD 00- | MONTHS AGO/miyezi  MORE THAN 24 MONTHS AGO /Kuposera miyezi 24/zaka ziwiri……………………………….……….…95  NOT SURE /Sakudziwa………………………………..98 | |
| Q24 | **Why was the ITN not re-treated?**  ***Ndi chifukwa chiyani simunanyike masikito anu mmankhwala?*** | No money to buy treatment kit/*Panalibe Ndalama zogulira mankhwala*………………..……………….1  No where to buy treatment kit/*Kulibe kogula mankhwala*………………………………………..…..2  It is long lasting treated net/*Ndiyothiridwa kale mankhwala*…………………………………………..…3  I just don’t want treated net/*sindimafuna masikito othira mankhwala*……………………….………………..……4 | |
| Q25 | **Did anyone sleep under this mosquito net last night?-**  Alipo wina aliyense yemwe anagona mumasikito usiku wathawu? | Yes/Inde………………………………….……..……1  No/Ayi ………….………………………...……..….2  Not sure/Sakutsimikiza…………………….……. …3 | |
| Q26 | **What shape of mosquito net do you prefer?**  **Mumakonda masikito otani?** | | Conical/yozungulira……………….1  Rectangle/yamakona…..…………..2  don’t know/ no preference /sakudziwa/iliyonse…………....….8 |
